# Supplementary material for: The Dual Prey-Inactivation Strategy of Spiders—In-Depth Venomic Analysis of Cupiennius salei
Source: Toxins (Basel). 2019 Mar 19;11(3):167. doi: 10.3390/toxins11030167 (PMC6468893; doi:10.3390/toxins11030167)
Supplement: Supplementary file 1 [file toxins-11-00167-s001.zip › Supplementary Fig S1.1-S1.19/Supplementary Fig S1.19.pdf]

Supplementary Fig S1.19

CLUSTAL O(1.2.4) multiple sequence alignment: Leucine-rich repeat domain-containing proteins

```
LRR_S1_CUPSA                -----VI-WKILLGLACLVASNVPECPPRENVYPCDCASDYYSTWIECRSIHGVEKMELA
LRR_ALOMA                   MLRYWVF-GKVFLGLVYLVISDVPECPPRENVYPCQCTNDHYFTSIGCTSVHGVDKLYPV
LRR_VIRFA                   -MRGWIF-GEVFLGLVCVGLSQVPNCPPSEDVYPCYCNNDYYLTSISCEGIGSTDKIEHV
tr_A0A087USK5_STEMI         ---MWFLKFFGIFGAASVILAGVPECPPSQKVYPCFCTNDGLLTSIDCEGLGSLQKISDV
LRR_OXYLI1                  -MHSWIF-WKIFLGLIYSVISGVPECPPRENVSPCQCTNDGFLTSVDCTGINGIDRLYPV
tr_A0A0S3Q1T5_HAELO         ------MSGLNLLNVLVVVVTLTPRV---LASPSCHDPSGTDYEHYRCFNFSSPDDFSRL
                                  :    ::.      : .*.         *   ..       * .. . : :

LRR_S1_CUPSA                LENT-RGLNVSYAFWKSRLGDIPSDFFKGQKSTSVHFENCQIGSFG---DRPFTGLEDTL
LRR_ALOMA                   LENT-KGLNVSYAFWKSRLGDIPSDFFKGQKSTSLHFENCQIGSFG---DRPFTGLENTL
LRR_VIRFA                   LKNT-RGLNMSYAFWKSKLGDIPSDLFKGQQSVNLHFENCKIGSFG---DRPFTGLEDTL
tr_A0A087USK5_STEMI         MKNI-KGQNLSFAFWKSNLGDIPSNFFEGFQSVNLHFENCKIGSFG---DRPFTGLENSL
LRR_OXYLI1                  LKNT-KGLNMSYAFWKSRLGDIPSNFFEGQQSVNLHFENCQIGSFG---DRPFTGLENSL
tr_A0A0S3Q1T5_HAELO         LERPQLHKDLHFVLKDSSLSHLPEGAFSQINASVLELNNVQLDVFNLEDENPLDGLQTSL
                            ::.     :: :.: .* *..:*.. *.  ::  :.::* ::. *.   :.*: **: :*

LRR_S1_CUPSA                KSIYI-YGSVDKRRKDLETFRLGHLKKVTELIFEANDIKRLGNDWFEGGPAPLKLLMLEA
LRR_ALOMA                   KDLYV-YGSVDKRRKDLESFRLGHLNKLQELIFEANDIKRLGNDWFEGGPAPLRLLMLEA
LRR_VIRFA                   KHLYI-YGSIDKRRKDLETFPLGHLKKLEYLAFQANDIKRLGNEWFEGGPSTLRQMMLEA
tr_A0A087USK5_STEMI         KNIYV-YGSVDKRKKDLETFPLGHLKKLKDLYFEANDIKRLGNDWFEGGPESLEQLMLEA
LRR_OXYLI1                  KRLYV-YGSVDKRKKDLETFRLGHLKKLEELIFEANDIKRLGNDWFEGGPAGITLLMLEA
tr_A0A0S3Q1T5_HAELO         RRLILGYGSTIPTS----WAPFSTLEKLTTVRISEAKDLV-LSRSFNELPPSVKVINVAF
                            : : : ***            :. *:*:  : :.  .     .  *:  *  :  : :

LRR_S1_CUPSA                NDIEELGDRAFASLENLEQIMLGDNRFGKISRSMFPRPATKLKYLETSFNGIRELPEDLF
LRR_ALOMA                   NDIEELGDRAFASLENLEQIMLGDNRFGKISRSMFPRPATKLRYLEASFNGIRELPEDMF
LRR_VIRFA                   NDIEEVGDRAFASLTNLEQIWMGDNRFKKVYRTMFPRPATKLQLLEMGFSEIEDLPEDMF
tr_A0A087USK5_STEMI         NDIEELGDKAFASLINMTQIWLGDNRFKKVSRSMFPNPANKLELLEMSFNGIEELPEDMF
LRR_OXYLI1                  NDIEELGDRAFASLVNLEQIWTGDNRYKKILRSMFPRPANKLRSIEISFNGIEELPEDFF
tr_A0A0S3Q1T5_HAELO         STIASVDEDWLSRLENLEVVGIRHCNLKVFQRSMLPRPAPKLWRLDLFKNELSSLPADFS
                            . * .:.:  :: * *:  :   . .   . *:*:*.** **  ::   . : .** *:

LRR_S1_CUPSA                EDMPVLNSINVSGNKLKSVPEATWGKIWGQLDEVYLERNEGFECDENIKWIYQRRLPRIL
LRR_ALOMA                   EDMPALNSINVSGNKLKSVPEATWGKIWDQLDEVYLERNEGFECDEKIKWIYQRRLPRIL
LRR_VIRFA                   EDMPSLTSINVAGNKLTSLPKATWGKIWDQLEEVYLERNKGFQCDDNMKWIYERKLPRIL
tr_A0A087USK5_STEMI         EGMPRLTSINVSGNKLKTVPEATWGKIWDQLNEVYLERNSGFVCDENMKWIYQRKLPKIL
LRR_OXYLI1                  EDMPALTSINVSGNKLKSVPEATWGKIWNQLDEVYLERNVGFQCDEKIKWIYKRRLPKIL
tr_A0A0S3Q1T5_HAELO         ADMPALRSVTVEHNQIKTFEEQTFAPLTNNDTNRVRFLGNPLHCDCKLRFVLSY-PPSWL
                             .** * *:.*  *::.:. : *:. : .:  :     .  : ** ::::: .   *  *
```

Percent Identity Matrix - created by Clustal2.1

		1	2	3	4	5	6
1:	LRR_CUPSA	100.00	85.90	69.87	67.95	75.64	22.19
2:	LRR_ALOMA	85.90	100.00	71.52	70.06	81.01	23.84
3:	LRR_VIRFA	69.87	71.52	100.00	71.02	70.25	21.85
4:	tr_A0A087USK5_STEMI	67.95	70.06	71.02	100.00	72.70	21.71
5:	LRR_OXYLI1	75.64	81.01	70.25	72.70	100.00	23.76
6:	tr_A0A0S3Q1T5_HAELO	22.19	23.84	21.85	21.71	23.76	100.00

Proteomic coverage trypsin

>LRR_S1_CUPSA leucine-rich repeat protein

MWYWVIWKIL LGLACLVAS**N VPECPPRENV YPCDCASDYY STWIECRSIH GVEKMELALE NTR**GLNVSYA FWK**SRLGDIP**
SDFFKGQKST SVHFENCQIG SFGDRPFTGL EDTLKSIYIY GSVDKRRKDL ETFRLGHLK**K VTELIFEAND IKRLGNDWFE**
GGPAPLKLLM LEANDIEELG DRAFASLENL EQIMLGDNRF GKISRSMFPR PATKLKYLET SFNGIRELPE DLFEDMPVLN
SINVSGNKLK SVPEATWGKI WGQLDEVYLE RNEGFECDEN IKWIYQRRLP RILTGQCRGG KFAGK**KLQDL TLADFD**

Proteomic coverage chymotrypsin

>LRR_S1_CUPSA leucine-rich repeat protein

MWYWVIWKIL LGLACLVAS**N VPECPPRENV YPCDCASDYY STWIECRSIH GVEKMELALE NTR**GLNVSYA FW**KSRLGDIP SDFFKGQKST SVHFENCQIG SFGDRPFTGL EDTLKSIY**IY GSVDKRRKDL ETF**RLGHLKK VTEL**IFEAND IKRL**GNDWFE GGPAPL**KLL**M LEANDIEELG DRAFASLENL EQIMLGDNRF GKISRSMFPR PATKL**KYLET SF**NGIRELPE DLFEDMPVLN SINVSGNKLK SVPEATWGKI WGQLDEVYLE RNEGFECDEN IKW**IY**QRRLP RILTGQCRGG KFAGKKLQDL TL**ADFD

Supplementary Fig S1.19 Multiple sequence alignment of LRR_S1 from *C. salei* (LRR_CUPSA [MH766648), *A. marikovskyi* (LRR_ALOMA), *V. fasciatus* (LRR_VIRFA), *O. lineatus* (LRR_OXYLY), *S. mimosarum* (tr_A0A087USK5_STEMI), and the tick *Haemaphysalis longicornis* (tr_A0A0S3Q1T5_HAELO). Signal peptides are marked in black characters, cysteines are marked in white characters with a black box, positively charged amino acids in pink, negatively charged amino acids in blue, hydrophobic amino acids in red and polar amino acids in green. The proposed leucine rich motifs are highlighted in yellow boxes.
